# Putative molecular targets for vitamin A in neutralizing oxidative stress in acute and chronic pancreatitis — a systematic review

**DOI:** 10.1007/s00210-023-02442-4

**Published:** 2023-02-27

**Authors:** Jacek Burzyński, Jakub Fichna, Aleksandra Tarasiuk

**Affiliations:** Department of Biochemistry, Medical University of Lodz, Mazowiecka 5, 92-215 Lodz, Poland

**Keywords:** Acute pancreatitis, Chronic pancreatitis, Oxidative stress, Vitamin A, Nrf2, MAPK, AMPK, TLR3, TLR4

## Abstract

Acute pancreatitis (AP) and chronic pancreatitis (CP) are debilitating diseases of gastrointestinal tract and constitute great threat for human health in high-income countries. Recent studies emphasize the impact of oxidative stress on development of these pathologies, and numerous authors evaluate the effect of the antioxidant therapy on the course of AP and CP. Though several antioxidative agents were discovered in the past decades, vitamins remain canonical antioxidants. Despite the fact that vitamin A is known for its antioxidative effect, there is little data about the impact of vitamin A on oxidative stress in the pathogenesis of AP and CP. The scope of the review is to evaluate molecular targets for vitamin A, which may be involved in oxidative stress occurring in the course of AP and CP. Our research of available literature revealed that several mechanisms are responsible for attenuation of oxidative stress in AP and CP, including Nrf2, MAPK, AMPK, TLR3, and TLR4. Furthermore, these factors are at least partially expressed in vitamin A-dependent manner, though further investigations are required for elucidating in detail the role of vitamin A in defense against reactive oxygen species. Our review revealed that vitamin A might influence the expression of several molecular pathways involved in antioxidative defense and cytoprotection; thus, its administration during AP and CP may change the course of the disease.

## Introduction

Inflammatory pathologies of the pancreas, including acute pancreatitis (AP) and chronic pancreatitis (CP), are the most common diseases affecting this organ and remain a major issue for healthcare system in high-income countries (Boxhoorn et al. 2020) AP is characterized by local and systemic inflammation that resolves within 1 week in majority of cases. Nevertheless, 20% of patients exhibit a more severe course of the disease or local and/or systemic complications, which may lead to distant organ failure or even death (20–40%) (Boxhoorn et al. 2020). On the contrary, in CP, persistent fibroinflammatory process occurs, resulting in irreversible damage of pancreatic parenchyma. Due to destruction of secretory structures, CP patients develop exocrine and endocrine pancreatic insufficiency, which exhibit as diabetes and/or malabsorption (Beyer et al. 2020) Despite the growth of medical knowledge and development of new therapeutical strategies, treatment of AP and CP remains poorly effective and mainly focuses on alleviating symptoms. As regards AP, fluid resuscitation and antibiotic therapy are commonly used, though in more severe course of disease, surgical intervention may be required. In terms of CP, treatment mainly involves administration of pancreatic enzymes (lipase, amylase, proteases) and/or insulin. Analgesics (metamizole, buprenorphine, tramadol) are essential part of management of both pathologies (Boxhoorn et al. 2020; Beyer et al. 2020).

Several mechanisms are involved in pancreatic damage, whether it occurs during acute or chronic process.

An early event in pathogenesis of AP is activation of NOD-like receptor family pyrin domain containing 3 (NLRP3), nuclear factor kappa-b (NFκB), and receptor interacting protein (Mayerle et al. 2019) (RIP3) receptors, which triggers cellular death, along with massive release of damage-associated molecular patterns (DAMPs) and inflammatory cytokines (such as interleukin-1β (IL-1β) and IL-6). Subsequently, macrophages and neutrophils infiltrate pancreatic tissue and secrete various enzymes (such as metalloproteinases, collagenases), leading to further destruction of acinar cells (Mayerle et al. 2019). Special attention is given to excessive accumulation of reactive oxygen species (ROS) in course of the pancreatitis. In physiological state, ROS are effectively scavenged by various endogenous factors (glutathione peroxidase (GPx), superoxide dismutase (SOD), or catalase (CAT)), though when inflammatory response is triggered, these mechanisms are insufficient, which results in oxidative damage of pancreatic tissue. Recently, numerous studies examined the impact of plant-derived compounds (Pohl and Lin 2018; Yarley et al. 2021; Anchi et al. 2017) on the course of oxidative damage; however, vitamins remain canonical exogenous antioxidants for mammals.

Though vitamin A (VA, retinol) was first described as an essential factor in the vision process, presently, its function extends beyond maintaining ocular homeostasis. It is estimated that VA regulates the expression of nearly 500 genes and exhibits anti-inflammatory, anticarcinogenic, and antiproliferative properties (Haymon 1957).

**Fig. 1:**
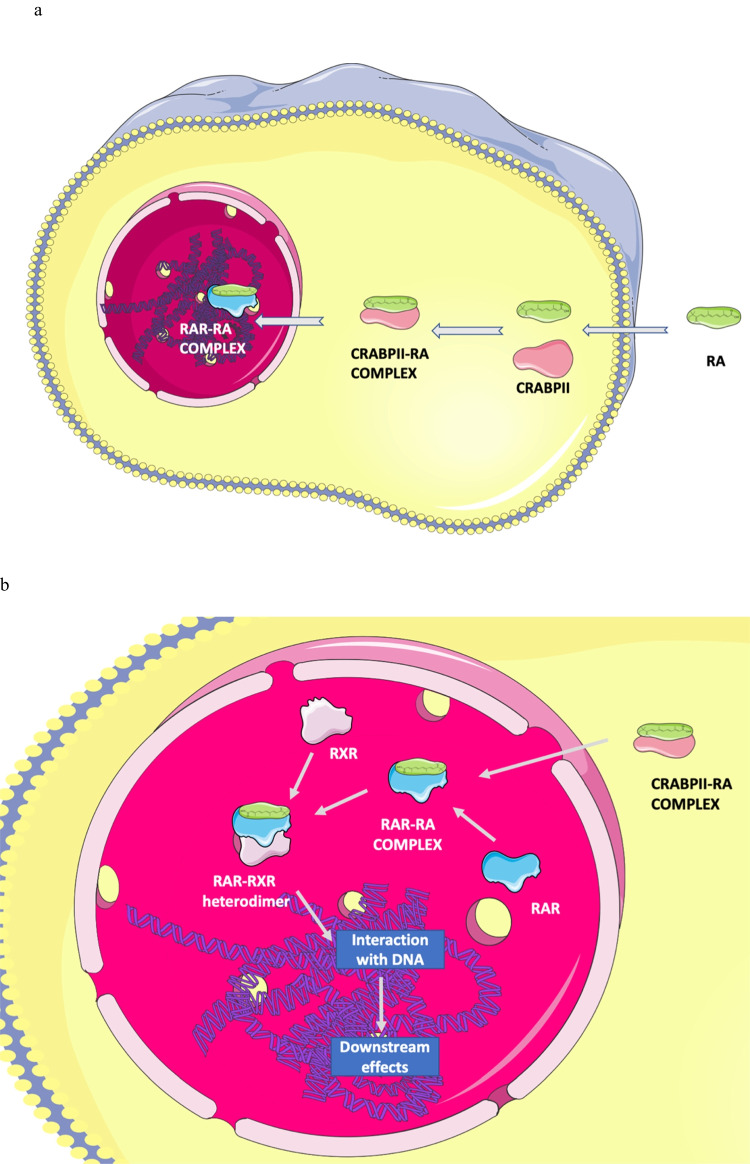
Retinoic acid signaling: **a** Transport of retinoic acid across membrane and cytoplasm. **b** Retinoic acid signaling in nucleus. RA retinoic acid, CRABPII cellular retinoic acid binding protein, RAR retinoic acid receptor, RXR retinoic X receptor

Retinoic acid is an active form of VA, which exerts its biological function by stimulation of transcriptional factors, so called retinoic acid receptors (RARs). As shown in Fig. 1, RA (as a fat-soluble compound) is transported directly through the cell membrane and in cytosol binds to cellular retinoic acid binding protein (CRABPII), which subsequently transfers RA to the nucleus. Within the nucleus, RA interacts with RAR to form a RA-RAR complex, which further binds to the retinoic X receptor (RXR), creating RAR-RXR heterodimers. These heterodimers directly interact with DNA and are responsible for the regulatory effects of RA (al Tanoury, Z., Piskunov, A. Rochette-Egly, C. 2013).

Despite the antioxidative properties of VA being widely described in literature (Haymon 1957; al Tanoury, Z., Piskunov, A. Rochette-Egly, C. 2013; Kim et al. 2021), there is little data on the influence of VA on oxidative stress (OS) occurring in the course of pancreatitis. Therefore, we conducted a systematic review, aimed to investigate molecular targets of VA, which may be involved in attenuating OS in AP and/or CP.

## Aim of the study

Due to limited amount of data about the impact of VA on OS in AP and/or CP, the aim of our study was to evaluate potential link between antioxidative properties of VA and OS in the pathogenesis of AP and/or CP.

## Methodology

We performed research of current literature published on PubMed, Scopus, and Google Scholar between January 2017 and April 2022. Our research was divided into two steps. Firstly, the databases mentioned above were searched with the following terms: vitamin A, acute pancreatitis, chronic pancreatitis, and oxidative stress to evaluate factors, whose expression is controlled in a VA-dependent manner and which are simultaneously involved in maintenance of redox homeostasis in AP and CP. Title and abstracts of collected papers were screened by JB under the supervision of AT. Once the putative factors were assessed, we extended our research by the name of the factor involved in neutralizing ROS, e.g., for the Nrf-2/KEAP1 pathway, the research included vitamin A, chronic pancreatitis, acute pancreatitis, and Nrf-2/KEAP1. In this step, we did not use time criteria. Afterwards, collected data were evaluated if they met inclusion criteria of our study.

In our research, we involved original papers, which were performed on cell culture of pancreatic acinar cells, animal models of AP or CP, and human pancreatic tissue. Several forms of VA were used in the analyzed studies, though we decided to include research evaluating properties of all-trans retinoic acid (ATRA), retinyl palmitate, and β-carotene due to their homogeneity of actions. However, studies investigating properties of crocetin, which is reported to have similar properties to VA, were excluded due to the not fully described mechanism of action of this compound.

## Results

### Keap1/Nrf2/ARE pathway

Kelch-like ECH-associating protein 1/nuclear factor erythroid 2-related factor 2-antioxidant response element (Keap1/Nrf2) signaling is a crucial element of antioxidative defense of cell, and alterations in this pathway are involved in the pathogenesis of pancreatitis. Nrf2 is a transcriptional factor, which regulates the expression of 250 genes involved in the cytoprotection and metabolism of xenobiotics. In the physiological state, Nrf2 is constantly degraded in Keap1-dependent manner. However, due to sensitivity of Keap1 to ROS, this process is altered during OS, resulting in stabilization of Nrf2 and its nuclear translocation. In the nucleus, Nrf2 binds to antioxidant-response element (ARE), leading to enhanced expression of endogenous antioxidants, such as heme-oxygenase 1 (HO-1) and NAD(P)H quinone dehydrogenase 1 (NQO1) (Cuadrado et al. 2019).

Numerous studies 11–14 revealed that Nrf2 expression is significantly downregulated during AP, whereas Fu et al. 15 reported that overexpression of Nrf2 enhanced the levels of SOD, HO-1, and NQO1 and mitigated AP in vivo and in vitro. Furthermore, our research of literature revealed that Nrf2 signaling was restored by several exogenous antioxidants, such as high-dose vitamin C, pomegranate extract, Tanshinone IIA (the main active component of red sage), and lycopene. However, this effect was abolished when inhibitors of Nrf2 were used (Xu et al. 2020; Gupta et al. 2019; Chen 2020; Lee et al. 2022). Nrf2 pathway may be influenced by several other factors, including melatonin, hepatocyte growth factor, vinpocetine, borneol, visnagin, chitosan oligosaccharides, or flavonoids derived from *Coreopsis tinctoria* nuts (Jung et al. 2010; Palestino-Dominguez et al. 2018; Abdelzaher et al. 2021; Bansod et al. 2021; Pasari 2019; Mei, et al. 2021; Du, et al. 2018). Unfortunately, these studies did not evaluate whether the alleviation of symptoms occurs once Nrf2 expression is blocked; thus, we suggest interpreting these results with caution. An interesting strategy was developed by Yao et al. (Yao et al. 2020). The authors developed nanoparticles containing complexes of silk fibroin and antioxidant bilirubin. These compounds were not active during physiological state, though when excessive activation of trypsin occurred, bilirubin was released from nanoparticles and attenuated OS in pancreas. The authors suggest that the antioxidative effect of bilirubin was exerted by direct neutralization of ROS along with enhancement of Nrf2 signaling.

The role of Nrf2 in pathogenesis of CP remains obscure. Choudhury et al. (Choudhury et al. 2015) reported that mice with lipopolysaccharide (LPS)-induced CP model had a significantly decreased activity of Nrf2 signaling. In compliance with this study, Yang et al. (Yang 2012) as well as Liang et al. (Liang 2021) reported a decrease in cellular level of Nrf2 in the mouse CP model, though L-cysteine and Dahuang Danshen Decoction (DDD) restored the Nrf2 expression.

Moreover, Nrf2 is a key factor in the pathogenesis of pancreatic ductal adenocarcinoma (PDAC), and it plays dual role in cancer development. In an early stage of carcinogenesis, diminished expression of Nrf2 increases ROS-driven DNA alteration, thus facilitating cancer development. However, during progression of PDAC, the high expression of Nrf2 is associated with increased cancer cell survival, resulting in enhanced cancer growth (Cykowiak and Krajka-Kuźniak 2022). Given that CP predisposes to PDAC (Hao et al. 2017), downregulation of Nrf2 expression in CP may be an essential step during progression from chronic inflammation to neoplasm.

Furthermore, Masuda et al. (Masuda et al. 2015) reported that induction of Nrf2 in beta cells resulted in increased survival due to enhanced expression of antioxidants, along with reduced secretion of inflammatory cytokines. In compliance with that, the study performed by Schultheis et al. (Schultheis 2019) revealed that activation of Nrf2 signaling restored physiological response of beta cells to glucose. These data indicate that activation of Nrf2 may be beneficial in patients with CP due to its positive effects on pancreatic islet cells homeostasis.

As regards the impact of VA on Nrf2 signaling, current literature remains inconclusive. Several studies reported that administration of VA led to restoration of Nrf2, along with mitigation of OS; however, the authors did not evaluate whether the antioxidative effect of VA was exerted exclusively through Nrf2 signaling (Wang et al. 2014; Cheng et al. 2019; Wu 2022; Latief et al. 2019). On the contrary, Yin et al. (Yin et al. 2015) reported that ATRA reduced the expression of Nrf2 and HO-1 in glial cells, resulting in enhanced inflammation and OS of brain tissue after intracerebral hemorrhage. Moreover, Jayakumar et al. (Jayakumar et al. 2015) used ATRA as inhibitor of Nrf2 signaling to examine the role of Keap1/Nrf2 pathway in DNA repair. Sapiro et al. (Sapiro et al. 2017) reported that ATRA slightly increased intracellular levels of Nrf2, though the cytoprotective effect of ATRA putatively arose independently to Nrf2 signaling.

A study performed by Xiu et al. (Xiu et al. 2007) seems to partially explain these discrepancies. The authors reported that the administration of ATRA did not influence the expression of Nrf2 and its nuclear translocation of Nrf2, though it activated retinoic acid receptor α (RARα), which subsequently altered downstream induction of antioxidant synthesis by Nrf2 pathway. Similar effect was observed regarding RAR-gamma; however, it was much less exacerbated. Unfortunately, the authors did not examine the impact of activation of other retinoic receptors such as RARβ, and it requires further investigation to entirely describe the impact of VA on the Keap1/Nrf2 pathway.

### MAPK pathway

The mitogen-activated protein kinase (MAPK) family is a large group of proteins, which are involved in intracellular signal transduction and control basic cellular functions such as differentiation, response to stress, and apoptosis (Plotnikov et al. 2011). Several authors reported that P38-MAPK signaling is enhanced during development of AP, and the inhibition of this pathway is associated with alleviation of its symptoms (Ma et al. 2018; Hu et al. 2019; Morsy and Ahmed 2020). Furthermore, An et al. (Cykowiak and Krajka-Kuźniak 2022) reported that OS may increase P38-MAPK signaling, whereas a study performed by Hu et al. 41 revealed that enhancement of the activity of this pathway is associated with decreased levels of endogenous antioxidants (SOD, GSH). These data indicate that OS may enhance P38-MAPK signaling pathway, which subsequently results in diminishing the antioxidative defense, leading to development of positive feedback loop. Moreover, a study performed by An et al. (An et al. 2020) revealed that OS induces P38-MAPK signaling and subsequently increases fibromodulin expression in the rat model of CP. Afterwards, it enhanced extracellular matrix (ECM) synthesis and activation of pancreatic stellate cells occurs, resulting in excessive fibrosis of the pancreatic tissue.

The impact of VA supplementation on P38-MAPK pathway is unclear. Few studies indicate that ATRA decreases P38-MAPK signaling (Pu 2022; Liu et al. 2018; Li et al. 2018), though Pu et al. (Pu 2022) reported that a greater effect on P38-MAPK activity was observed when L-cysteine (a strong antioxidant) was administered. This indicates that ATRA may not directly interact with P38-MAPK, and the decrease in its activity is a result of alleviation of OS. On the contrary, Namachivayam et al. (Namachivayam et al. 2015) reported that ATRA increases the activity of P38-MAPK. In conclusion, given that attenuation of OS alone may decrease the P38-MAPK activity, it is crucial to establish whether VA mitigates the activity of this pathway by modulating OS alone or if there is any mechanism explaining the impact of VA on P38-MAPK signaling.

### TLR4

Toll-like receptor (Pohl and Lin 2018) (TLR4) signaling plays a crucial role in the development of both AP and CP. As a result of cell necrosis, several DAMPs are released, which consequently trigger the activation of TLR4 signaling, leading to exacerbation of inflammation and OS. In terms of CP, administration of transforming growth factor β-activated kinases (TAK-242), a TLR4 inhibitor, resulted in reduced synthesis of extracellular matrix and organ fibrosis (Pan et al. 2017).

In OS, an enhanced expression of TLR4 signaling induced oxidative damage in the pancreatic tissue. Pan et al. (Pan et al. 2016) reported that excessive accumulation of ROS occurred in acinar cells overexpressing TLR4. Furthermore, administration of TAK-242 resulted in the mitigation of OS induced by taurocholate. A decrease in lipid peroxidation and the restoring of mitochondrial homeostasis were observed as well. A study conducted by Hong et al. (Hong 2020) revealed that high-lipid diet induces expression of TLR4 and OS, though these effects were diminished by the administration of TAK-242. Moreover, Xie et al. (Xie et al. 2021) reported that Prussian blue nanozymes (PBzyme) significantly scavenged ROS and alleviated OS in the course of AP. Furthermore, in the same study, potential molecular targets of PBzyme were investigated, and the authors revealed that besides direct neutralization of ROS, PBzyme exerted antioxidative and anti-inflammatory effect via inhibition of TLR4/NFκB pathway.

Analysis of current literature revealed that VA may inhibit TLR4 signaling. Li et al. (Li et al. 2017) reported that administration of ATRA resulted in diminished TLR4 expression, along with enhanced macrophage phagocytosis in acute lung injury. Moreover, administration of ATRA mitigated diabetic nephropathy in a TLR4-dependent manner (Sierra-Mondragon et al. 2018). In compliance to these results, Young et al. (Kim et al. 2013) reported that retinol inhibited the synthesis of downstream targets of TLR4. As regards intestinal inflammation and epithelial integrity, current data is inconclusive. Cheng et al. (Cheng et al. 2021) reported that administration of β-carotene alleviated LPS-induced inflammation and disturbance in epithelial integrity in a TLR4-dependent manner, whereas ATRA did not exert similar effect. On the contrary, a study performed by Li et al. (Li et al. 2017) reported that treatment with ATRA resulted in an enhanced TLR4 expression in RAR-β dependent manner, which was linked with improvement of intestinal integrity. Thus, further studies should focus on the impact of activation of different RARs on TLR4 signaling, along with influence of various VA derivatives on this pathway.

### TLR3

In contrast to TLR4, the activation of TLR3 seems to alleviate symptoms of AP. Huang et al. (Huang et al. 2019) reported that administration of polyinosinic/polycytidylic acid (polyI:C), a selective TLR3 activator, resulted in enhanced interferon-β (IFN-β) secretion, along with diminished adhesion and invasion of neutrophils. Furthermore, activation of TLR3 mitigated oxidative damage in the pancreatic tissue.

A study performed by Bernardo et al. (Bernardo et al. 2013) revealed that the co-administration of retinoic acid (RA) and T polyI:C resulted in synergic upregulation of IFN-dependent apoptosis in breast cancer cells. The observed effect was significantly diminished when only RA or polyI:C was administered. Similarly, Szabo et al. (Szabo et al. 2012) reported that ATRA and polyI:C acted synergically to enhance chemokine and IFN-β secretion in melanoma cell culture (WM35, WM983A). However, studies conducted by Kim et al. (Kim et al. 2013) and Pu et al. (Pu 2022) indicated that the administration of ATRA or RA downregulated TLR3 synthesis; thus, the exact impact of VA on TLR3 signaling remains unclear.

### AMPK

Though 5′AMP-activated protein kinase (AMPK) signaling pathway is essential in maintaining redox homeostasis, its role in defense against ROS in pancreatitis remains obscure (Ren and Shen 2019). Srinivasan et al. (Srinivasan et al. 2021) reported that AMP activity was diminished by administration of ethanol to pancreatic acinar cells, resulting in enhanced OS and production of inflammatory cytokines. Another study performed by the same authors revealed that treatment with AICAR, an AMPK-activator, diminished ROS accumulation in pancreatic acinar cells (Srinivasan 2021). In compliance with these results, a study conducted by Bansod et al. (Bansod et al. 2020) indicated that berberine (BRB), a natural alkaloid, exerted anti-inflammatory and antioxidative effect in CP through an AMPK-dependent manner. Moreover, Tarasiuk et al. (Tarasiuk et al. 2019) reported that administration of BRB alleviated inflammation in the pancreas and the lungs in the course of AP. These data indicate that BRB may be potential therapeutic agent in treatment of AP and should be further evaluated in CP.

Current literature indicates that VA may induce AMPK signaling. Kim et al. (Kim et al. 2015), along with Ishijima et al. (Ishijima et al. 2015), reported that administration of RA resulted in enhanced AMPK activity. Furthermore, a study performed by Yun et al. (Yun et al. 2008) revealed that RA stimulated glucose uptake in an AMPK-dependent way.

## Conclusions

Despite the great impact of OS in pathogenesis of AP and CP, mechanisms leading to alleviation of OS in pancreas remain poorly described in the current literature. The authors mainly focus on Nrf2 signaling, as a canonical antioxidative agent, and discuss in what manner it is influenced by various exogenous antioxidants. Unfortunately, only a few studies examined whether the observed antioxidative effects and mitigation of symptoms of AP and CP result from an exclusive stimulation of Nrf2 pathway (Xu et al. 2020; Gupta et al. 2019; Chen 2020; Lee et al. 2022). Furthermore, the impact of other signaling pathways on oxidative damage of pancreas is much less evaluated compared to Nrf2 signaling. Though experimental studies suggest that alleviation of OS may improve symptoms of AP and CP, results of clinical trials do not support this hypothesis. It was reported that administration of antioxidants is associated with improved quality of life and diminished use of analgesics in CP patients, yet no impact on exo- and endocrine functions was reported (Gao, et al. 2021). Akin to CP, antioxidant therapy (e.g., with VA) in patients with severe AP did not influence the course of disease in terms of mortality, severity of disease, and organ dysfunction. However, it should be emphasized that VA is not a typical antioxidant and besides direct neutralization of ROS, it regulates transcription of numerous genes and influences several molecular pathways. Noteworthy, it was reported that RA signaling increases during inflammation of pancreas and is associated with tissue repair. Given that VA exerts pleiotropic function in the organism, including antioxidant and anti-inflammatory effects, it is possible that patients suffering from AP or CP will benefit from VA-based therapy.

Our review revealed that TLR signaling may be crucial in the antioxidative defense, as well as AMP and MAPK pathways; thus, these factors might become potential therapeutic targets in treatment of AP and/or CP. Moreover, the authors mentioned that several different factors, such as NLRP3, PI3K/AKT/mTOR, or NFκB, are involved in the pathogenesis of AP and/or CP, though a detailed correlation between them and OS was not evaluated (Xue, et al. 2019; Kong et al. 2021; Tarasiuk et al. 2021). Moreover, given that the factors mentioned above are involved in regulating cellular death, further studies examining the impact of OS on their expression and its influence on cellular death would by highly interesting.

Several authors reported conflicting results about the impact of VA on OS; thus, it is difficult to predict its influence on the pathogenesis of AP and/or CP (Table 1).

**Table 1:** Summary of putative targets of vitamin A in alleviating oxidative stress in the course of AP and CP

Name of the factor	Impact on OS	Change of expression		Influence of VA on the expression of the factor	References
		Pathogenesis of AP	Pathogenesis of CP		
Keap1/Nrf2	Induction of the synthesis of endogenous antioxidants	Decrease	Decrease	Increase Decrease	Schultheis 2019; Wang et al. 2014; Cheng et al. 2019) Wu 2022; Latief et al. 2019; Yin et al. 2015; Jayakumar et al. 2015)
P38-MAPK	Diminishment of the synthesis of endogenous antioxidants	Increase	Increase	Increase Decrease	Li et al. 2018) Pu 2022; Liu et al. 2018; Li et al. 2018; Namachivayam et al. 2015; Pan et al. 2017; Pan et al. 2016; Hong 2020; Xie et al. 2021; Li et al. 2017; Sierra-Mondragon et al. 2018; Kim et al. 2013; Cheng et al. 2021; Huang et al. 2019; Bernardo et al. 2013; Szabo et al. 2012; Ren and Shen 2019; Srinivasan et al. 2021; Srinivasan 2021; Bansod et al. 2020; Tarasiuk et al. 2019; Kim et al. 2015; Ishijima et al. 2015; Yun et al. 2008; Gao, et al. 2021; Xue, et al. 2019; Kong et al. 2021)
TLR4	Induction of the accumulation of ROS	Increase	Increase	Increase Decrease	Xie et al. 2021) Li et al. 2017; Sierra-Mondragon et al. 2018)
TLR3	Alleviation of OS	Unknown	Unknown	Increase Decrease	Huang et al. 2019; Bernardo et al. 2013) Sierra-Mondragon et al. 2018; Kim et al. 2013; Cheng et al. 2021; Huang et al. 2019; Bernardo et al. 2013; Szabo et al. 2012; Ren and Shen 2019; Srinivasan et al. 2021; Srinivasan 2021; Bansod et al. 2020; Tarasiuk et al. 2019; Kim et al. 2015; Ishijima et al. 2015; Yun et al. 2008; Gao, et al. 2021; Xue, et al. 2019; Kong et al. 2021)
AMPK	Alleviation of OS	Decrease	Decrease	Increase	Tarasiuk et al. 2019; Kim et al. 2015; Ishijima et al. 2015)

*AMPK* 5’AMP-activated protein kinase, *AP* acute pancreatitis, *CP* chronic pancreatitis, *Keap1/Nrf2* Kelch-like ECH-associated protein 1/nuclear factor (erythroid-derived 2)-like 2, *MAPK* mitogen-activated protein kinase, *OS* oxidative stress, *ROS* reactive oxygen species, *TLR* toll-like receptor, *VA* vitamin A.

Our review revealed that VA may influence molecular pathways involved in the maintenance of redox homeostasis in the pancreas, though there is not enough data to establish whether the administration of VA may lead to mitigation of symptoms of AP and/or CP. However, little is known about the exact mechanisms of action of VA, and only few are emphasized. VA exerts its antioxidative and protective effects through activation of specific RAR such as RARα, RARβ, and RXR. It can be hypothesized that VA may exert different effects depending on the type of RAR, which is dominantly expressed in the particular tissue.

In summary, our review indicates that VA may influence OS in the course of inflammatory states of pancreas, though it is difficult to predict particular outcome of VA administration. Given that current literature describes VA as an antiproliferative, anti-inflammatory, and antioxidative agent, it seems that VA should alleviate symptoms of AP and CP; however, some conflicting results were reported. To fully elucidate the impact of VA on OS in AP and CP, further studies with well-designed methodology are required. It is important to evaluate specific receptors for RA and its derivatives which are involved in VA downstream effects. Furthermore, given that OS itself influences various pathways, it is crucial to determine whether the impact of VA on a given pathway results from its direct interaction or attenuation of OS in different mechanism. Therefore, a holistic approach is required to fully elucidate molecular downstream effects of VA and to avoid potential bias.

## Data Availability

Not applicable.

## Abbreviations

AMPK: 5'AMP-activated protein kinase
AP: Acute pancreatitis
ARE: Antioxidant response element
ATRA: All-trans retinoic acid
BRB: Berberine
CP: Chronic pancreatitis
DAMPs: Damage-associated molecular
HO-1: Heme oxygenase 1
IFN: Interferon
IL: Interleukin
Keap1/Nrf2: Kelch-like ECH-associated protein 1/nuclear factor (erythroid-derived 2)-like 2
LPS: Lipopolysaccharide
MAPK: Mitogen-activated protein kinase
NFκB: Nuclear factor kappa-b
NLRP3: NOD-like receptor family pyrin domain containing 3
NQO1: NAD(P)H quinone dehydrogenase 1
OS: Oxidative stress
PDAC: Pancreatic ductal adenocarcinoma
polyI:C: Polyinosinic:polycytidylic acid
RA: Retinoic acid
RAR: Retinoic acid receptor
RIP3: Receptor interacting protein 3
ROS: Reactive oxygen species
SOD: Superoxide dismutase
TAK-242: Transforming growth factor β-activated kinases
VA: Vitamin A
